# Correlating Wine Quality Indicators to Chemical and Sensory Measurements

**DOI:** 10.3390/molecules20058453

**Published:** 2015-05-12

**Authors:** Helene Hopfer, Jenny Nelson, Susan E. Ebeler, Hildegarde Heymann

**Affiliations:** 1 Department of Viticulture & Enology, University of California-Davis, One Shields Ave., Davis, CA 95616, USA; E-Mails: hhopfer@ucdavis.edu (H.H.); jenny_nelson@agilent.com (J.N.); seebeler@ucdavis.edu (S.E.E.); 2 Food Safety & Measurement Facility, University of California-Davis, One Shields Ave., Davis, CA 95616, USA; 3 Agilent Technologies, Inc., 5301 Stevens Creek Blvd., Santa Clara, CA, 95051, USA

**Keywords:** wine quality, Cabernet Sauvignon, descriptive analysis, volatile analysis, elemental analysis, quality proxies

## Abstract

Twenty-seven commercial Californian Cabernet Sauvignon wines of different quality categories were analyzed with sensory and chemical methods. Correlations between five quality proxies—points awarded during a wine competition, wine expert scores, retail price, vintage, and wine region—were correlated to sensory attributes, volatile compounds, and elemental composition. Wine quality is a multi-faceted construct, incorporating many different layers. Depending on the quality proxy studied, significant correlations between quality and attributes, volatiles and elements were found, some of them previously reported in the literature.

## 1. Introduction

The quality of wine is difficult to define, as it is a multi-faceted construct, lacking a uniform and generally accepted definition. This is most certainly accredited to everyone’s different perception of quality. Due to this subjective layer of quality, authors such as Charters & Pettigrew [1] instead measure the perception of wine quality, and study how this perception differs among different populations. This holistic approach incorporates, therefore, all different aspects of wine quality, including the so-called extrinsic and intrinsic factors of quality [1,2]. For wine consumers, however, the question remains of how to select a bottle of quality wine? Thach [3] showed that consumers seek the advice of wine experts and/or other trusted sources, followed by other proxies of wine quality such as price, geographical origin, and age. Other wine quality proxies would be the absence of common wine defects (e.g., high levels of acetic acid, cork taint), and levels of the defect-causing compounds can be limited by governmental agencies. For example, wines from Austria can only be sold as *Qualitätswein (quality wine)* if they pass both a chemical and sensory assessment, are made from certain permitted varieties, and come from specified geographic regions [4]. However, these assessments are covering the lower end of wine quality, and leave much room for different levels of wine quality above this minimum level.

Despite the different quality perceptions, a generally accepted and less subjective quality baseline could be established by linking sensory and chemical measurements to wine quality. In order to establish such a baseline, different existing quality proxies, such as retail price, geographical origin, wine judgment medals and expert scores should be studied through the correlation with analytical measurement for their stability and ability to consistently measure wine quality.

One quality proxy is the retail price; assuming that a certain level of quality implies certain product costs that need to be covered by the wine price. In experiments with both wine novices and wine experts [5], the perceived quality of wine correlated significantly positively with the price consumers were willing to pay, however, the correlation between price and quality was higher for the wine experts than for the wine novices (0.63 *vs*. 0.46; both *p* < 0.01). Hence, higher quality wines are to a certain extent also higher in price. However, the final bottle price includes also distribution and retail costs, and cost of production is only a fraction of the total costs of a bottle of wine. Nevertheless, increased production costs will also be reflected in the final product costs.

The geographical origin of wine is another quality proxy. All around the world certain regions are known for their wines, and are considered high in quality, making that particular region famous for its wines. In order to maintain their reputations, many wine regions nowadays also require certain production and quality standards in order to label the wine with a regional label. In this instance, wine quality is associated with regionality or regional typicality, although the measurement or even the definition of typicality is as vague as the term of quality. The challenge is how to measure typicality; what makes a regional product special compared to products made under identical conditions from other areas?

One attempt to measure regionality is the measurement of the elemental fingerprint, *i.e.*, the elemental composition. Kelly *et al.* summarized that such measurements are based on the assumption that “… the vegetation is the compositional reflection of the bioavailable and mobilized nutrients present in the underlying soils from which they were cultivated. […] Consequently, the range of soils present and bioavailability mean that elemental composition may provide unique markers in food that characterise geographical origin.” [6] (p. 558)*.* Studies on the determination of geographical origin based on multi-elemental fingerprint are numerous for wines, and have compared wines from different regions within one country and between different countries, both in the old and new wine world [7,8,9,10,11,12,13,14,15,16,17,18,19,20]. Lately, research is also tackling the questions of defining a baseline, assessing the product variability within the region compared to outside the region in combination with the impact of winemaking (e.g., [21]). It is accepted that elemental fingerprints could be used for determining geographical origin [6]; therefore, the measurement of the elemental composition of wine could also serve as another wine quality proxy—a proxy for regionality.

In blind tastings (*i.e.*, without any extrinsic factors such as brand, price *etc*. available) wine consumers decide solely based on the intrinsic tasting experience. It was shown that flavor is the primary proxy for overall wine quality, and the importance of flavor on wine quality is undisputed [1]. Flavor as a multisensorial construct that incorporates ortho- and retro-nasal aromas, taste, and mouthfeel sensations into one flavor perception in the human brain is shown to be the main driver for overall quality perception [1]. As the aroma of wines is composed of complex mixtures of volatile compounds, gas chromatography with mass spectrometry is the primary choice for wine aroma analysis, and has been applied to solve questions about wine aroma composition (e.g., the effect of wine blending [22], and wine storage and packaging [23,24] to name only a few). The measurement of wine aroma profiles therefore provides another way of assessing wine quality—are there certain volatiles linked to quality scores and sensory attributes associated with wine quality?

In summary, wine quality is a multi-faceted construct, encompassing many different layers. In this work, we used an inter-disciplinary and cross-platform approach to further the understanding of wine quality. We combined descriptive sensory science with the chemical analyses of volatiles and elemental composition to link wine quality proxies to instrumental measurements of elemental and flavor composition, and sensory attributes, using a well defined set of 27 commercial Californian Cabernet Sauvignon wines.

## 2. Results and Discussion

### 2.1. Correlations of Wine Quality Parameters to Each Other

In an initial step, the relationships among the five chosen wine quality proxies were studied. Of all wine quality indicators (vintage, region, bottle price, wine points from wine competition, expert scores), only the points from the wine competition and the expert scores correlated significantly with each other (*r*(25) = 0.41, *p* < 0.05). In contrast to other research (e.g., [5]), retail price did not correlate with the points or the expert scores. However, this could be explained by the different study design between our study and the work by D’Alessandro & Pecotich [5]. In the previous study, wine consumers (novices and experts) tasted the wines and were then asked for the bottle price they were willing to pay. In contrast, in our study, the retail prices were set by the producing wineries, and our wine consumers tasted all wines in a blind setting and had no knowledge of or influence on the retail price. One could speculate that in our study retail prices would have been either accepted or dismissed by the consumers, based on their quality assessment if prices would have been revealed after the tasting. This, however, was not the purpose of our work; we were interested in the intrinsic quality perception, independent of any extrinsic factors such as retail price.

### 2.2. Sensory Profiling

**Figure 1:**
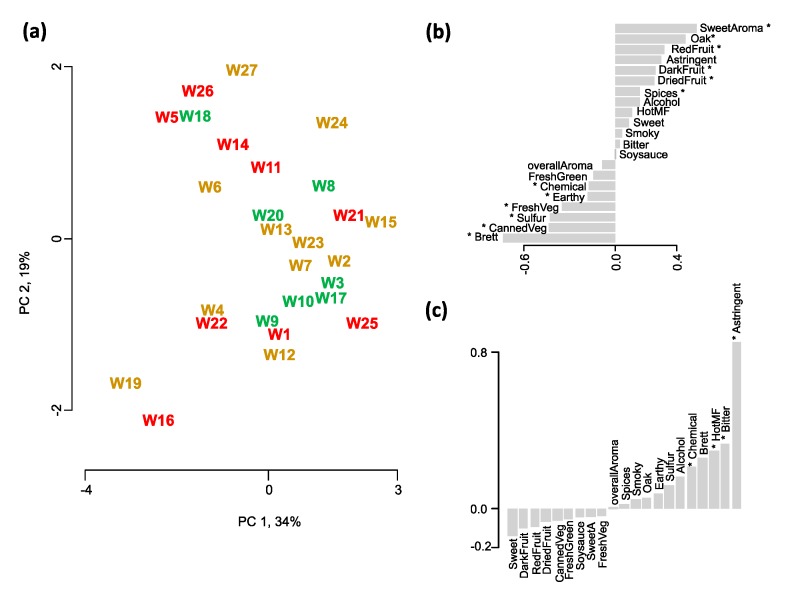
PCA of the sensory attributes that differed significantly (*p* < 0.05) in the ANOVA among the 27 wine samples. (**a**) Score plot showing the sensory space of the wines. Wines are color-coded according to their assigned quality categories based on the wine judgment—red for wines low in quality, (*i.e.*, that were awarded no medal), dark yellow for medium quality (*i.e.*, either a bronze or silver medal), and green for high quality (*i.e.*, a gold or double gold medal). Barplots of sensory attributes for (**b**) the first principal component (PC 1), and (**c**) the second principal component (PC 2). Attributes that are significantly correlated to either one of the first two PCs are denoted with an asterisk (*p* < 0.05).

Wine flavor is without a doubt a very important wine quality indicator. For the elucidation of the differences in wine flavor among the 27 studied wines, a trained sensory panel evaluated all the wines as described in [25]. The panel used 27 aroma, taste and mouthfeel attributes to describe the perceived sensory differences among the wines. Of these attributes, 21 differed significantly among the wines (17 aroma terms: overall aroma, alcohol, Brett (*i.e.*, aromas reminiscent of medicinal, leather, horse sweat, or barnyard, depending on the concentration and strain of the wine spoilage yeast *Brettanomyces bruxellensis*), canned vegetable, chemical, dark fruit, dried fruit, earthy, fresh green, fresh vegetable, oak, red fruit, smoky, soy sauce, spicy, sulfur, sweet aroma; two taste terms: sweet, bitter; two mouthfeel terms: astringent, hot), using analysis of variance (ANOVA) at a significance level of 5%. These significantly different attributes were used in a principal component analysis (PCA) to display the sensory differences among the 27 wines shown in Figure 1. Using the Kaiser criterion (*i.e.*, all dimensions with eigenvalues above 1) and the scree test (*i.e.*, observation of a “knee” when plotting the eigenvalues over the dimensions) the first two principal components (PCs) were kept, explaining 53% of the total variance. Wines were separated along the first principal component (PC 1), explaining 34% of the total variance, due to the large differences in oak and fruit compared to chemical and green aromas. Wines positioned on the left of the PCA score plot (Figure 1a) were rated high in descriptors that are associated with microbial and/or chemical spoilage (e.g., Brett, sulfur and chemical), and low in oak, sweet and various fruit aromas. Wines are color-coded according to the quality categories assigned in the wine competition (low quality, medium quality, high quality), but no separation due to wine quality is apparent along PC 1, as high quality wines are located next to low quality wines. It seems that the wine judges did not similarly score wines with very similar flavor profiles.

Along PC 2, explaining another 19% of the total variance, mouthfeel and taste differences together with some aromas contribute to the separation of the wines. Wines W17, W24, W20, and W5 scored higher in astringency, and lowest in fruit aromas and sweet taste. Again, no separation of the wines due to their quality categories is apparent.

To study if individual sensory descriptors indicate high or low quality, correlations of each sensory descriptor to the various quality indicators—points awarded in the wine competition (“points”), geographical origin of the vineyards (“regions”), wine vintage (“vintage”), retail bottle price (“price”), and expert liking scores (“experts”)—were carried out.

None of the sensory descriptors correlated significantly to the points awarded at the wine competition, while the wine expert ratings showed significant negative correlations to the aromas of soysauce (*r*(25) = −0.55, *p* < 0.05), and fresh green (*r*(25) = −0.41, *p* < 0.05). This could be explained two-ways. In the first explanation, judges at the wine competition did not use similar sensory frameworks when judging the wines and/or were not consistent in their assessment of quality, while the wine experts showed more agreement in their judgment. This explanation is supported by the studies by Hodgson [26] and Gawel *et al.* [27]. It is also supported by the fact that no descriptive framework was used in the wine competition. Judges were asked to rank tasted wines according to their individual criteria, and no system for judges’ alignment was used. Therefore, individual differences in quality perception are most certainly contributing to the final points awarded to the wines. The second explanation could be that (high) quality is not driven by individual sensory descriptors, but is the result of several descriptors acting together. This would explain that only negative correlations were found between sensory descriptors and the expert scores—experts have a common understanding of low quality, but differ in their high quality assessment. In our previous work [25], we found that wine experts use a quality framework that combines both descriptive terms and more subjective, personal preferences. Although there are personal differences among the experts, a common baseline exists for low-quality wines. In two open-ended questions (*Which attributes do you associate with a high quality wine?* and *Which attributes do you associate with a low quality wine?*), experts associated low wine quality with the presence of defects and flaws, such as microbial spoilage, presence of atypical aromas (e.g., vegetal-green) or oxidation aromas, or an unbalanced flavor profile [25]. It seems that the descriptors soysauce (*r*(25) = −0.55, *p* < 0.05), fresh green (*r*(25) = −0.41, *p* < 0.05), and overall aroma (*r*(25) = −0.65, *p* < 0.05) fall into these categories, thus, explaining their significant negative correlation to the expert scores. For high quality, the experts named the presence of fruit aroma as an important component of wine quality, therefore, it not surprising that red fruit aroma showed a significant positive correlation to the experts’ scores (*r*(25) = 0.45, *p* < 0.05).

For bottle price, six sensory attributes showed significant positive correlations. Bitter taste (*r*(25) = 0.53, *p* < 0.05), hot mouthfeel (*r*(25) = 0.59, *p* < 0.05), astringent mouthfeel (*r*(25) = 0.40, *p* < 0.05), alcoholic aroma (*r*(25) = 0.40, *p* < 0.05), and Brett aroma (*r*(25) = 0.48, *p* < 0.05), all showed a positive correlation to bottle price. The price of a bottle of wine reflects to a certain extent the costs of producing this bottle. Wines that are harvested later at higher sugar levels are typically higher in ethanol content, and the higher ethanol leads to higher perceivable alcoholic aroma and hot mouthfeel [28]. Increasing sugar content in grape berries can be accomplished by reducing competition for sugar allocation and improving sunlight exposure, *i.e.*, leaving fewer berry clusters on each vine, or reducing the leaf cover to increase sunlight exposure. All these practices increase vineyard management costs. Similarly, this is true for astringency and bitterness, the sensory response to polyphenols, mainly tannins, present in the wine [29]. Tannins in wine come from the grape berries (seed, skin, stem tannins), from enological tannin additions or from oak barrels, which in turn increase again the production costs [30]. The correlation between bottle price and Brett aroma is less intuitive to explain, and might be the result of the wine set used in this study. Typically, the presence of Brett aroma is considered at least an unwanted, if not even faulty, aroma [31]. One possible explanation could be oak barrels infected with Brettanomyces strains. Due to their high costs, oak barrels are typically re-used, difficult to properly sanitize, and provide with a porous surface, small oxygen ingress, and available cellobiose ideal conditions for Brettanomyces colonization [32,33,34], which can lead to detectable Brett aromas in the stored wines.

Red fruit aroma showed a negative correlation to bottle price (*r*(25) = −0.42, *p* < 0.05) which could be explained by the positive correlation to alcoholic aroma and hot mouthfeel—increasing ethanol content has previously been shown to decrease the perception of fruity aromas [28,35].

Three sensory attributes correlated significantly to vintage: astringent mouthfeel (*r*(25) = −0.59, *p* < 0.05), overall aroma (*r*(25) = −0.48, *p* < 0.05), and chemical aroma (*r*(25) = −0.45, *p* < 0.05) showed a negative association with wine age. With increasing age, polyphenols responsible for astringency polymerize and decreased in impact [36]. Similarly, compounds that were associated with chemical aroma (in this study, the verbal description was *the smell of ammonia and chlorinated swimming pool*) were not detected in older wines, either because they were never present or they decreased over time in the bottle.

Lastly, two sensory attributes showed significant differences among the nine geographical wine regions (Table 1). Sweet taste was rated significantly higher in the Lodi/Woodbridge region (*r*(25) = 0.47, *p* < 0.05) compared to all other regions. Looking at the average growing degree days (GDDs) the Lodi area shows the second highest number of GDDs, only surpassed by the most southern wine region in California (region G). Fresh green aroma (*r*(25) = 0.56, *p* < 0.05) was significantly higher in the coast regions E and G, and the Lodi area. The significantly higher perceived sweetness in the wines from the Lodi/Woodbridge region could be attributed to higher residual sugar levels or to the higher GDDs. All wines were considered dry (less than 1 g/L fermentable sugars), but interestingly, wines from the Lodi region had the highest levels of ethanol (15.4% (v/v) vs. the next highest levels of 15.1% (v/v) for region C (Napa County, CA, USA); data not shown).

**Table 1:** Mean ratings of two sensory attributes, fresh green aroma and sweet taste, differ significantly among the nine wine regions in California, USA. Mean ratings in the same column sharing a common lowercase letter are not significantly different from each other (*p* < 0.05). Mean values are calculated from two to four different wines per region and three sensory replicates for each wine.

Region	Region Code	Fresh Green Aroma	Sweet Taste
North Coast	A	1.0 c	1.7 bc
Sonoma County	B	1.3 bc	1.3 c
Napa County	C	1.0 c	1.7 bc
Greater Bay area	D	1.0 c	1.7 bc
North Central Coast	E	1.8 ab	1.8 bc
South Central Coast	F	1.3 bc	2.0 bc
South Coast	G	2.1 ab	1.7 bc
Sierra Foothills	H	1.5 b	1.7 bc
Lodi/Woodbridge	I	1.6 ab	2.7 a

The perception of fresh green aromas (in this study the corresponding reference standards included herbal, fresh cut grass and minty) could be related to the viticultural practices and growing conditions [34]—no correlation to GDDs is apparent as region G shows the highest number of GDDs while region E shows the lowest number (5621 *vs*. 2919, see Table 6).

### 2.3. Volatile Profiling

**Figure 2:**
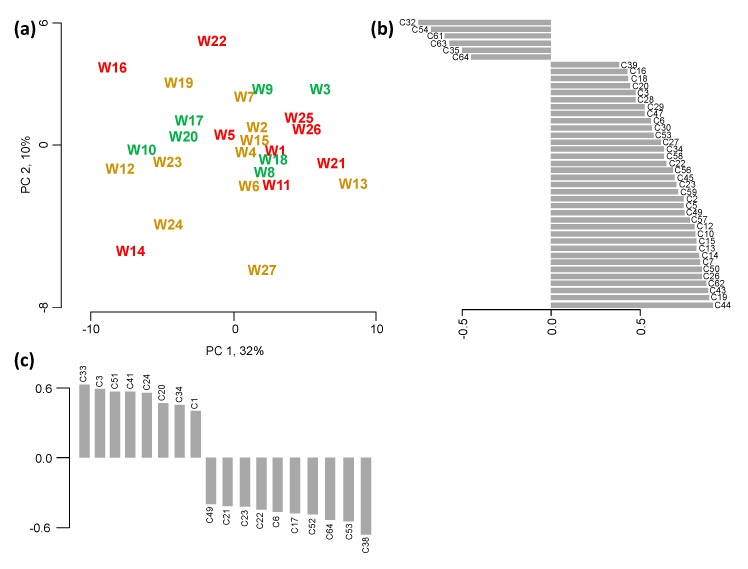
PCA of the volatile compounds that differed significantly among the 27 wines (*p* < 0.05). (**a**) Score plot showing the volatile space of the wines. Wines are color-coded according to their assigned quality categories based on the wine judgment—red for wines low in quality, (*i.e.*, that were awarded no medal), dark yellow for medium quality (*i.e.*, either a bronze or silver medal), and green for high quality (*i.e.*, a gold or double gold medal). Barplots of volatile compounds that contributed significantly (*p* < 0.05) to the separation (**b**) along the first principal component (PC 1), and (**c**) along the second principal component (PC 2).

A total of 64 volatile compounds (Table 7) were detected in the 27 wines using the described headspace solid-phase microextraction-gas chromatography-mass spectrometry (HS-SPME-GC-MS) method. Of these 64 volatiles, only one compound (C31, methyl hexanoate) did not differ significantly among the wines (*p* < 0.05) as determined by ANOVA. All significant correlations (*p* < 0.05) between sensory descriptors and volatile compounds are summarized in Table 2. All significantly different compounds were used in the PCA to obtain the volatile space of the studied wines, shown in Figure 2. Again, the Kaiser criterion and the scree test were used to decide how many PCs to retain. The first two principal components (PCs) were kept, explaining 42% of the total variance. With the exception of W3 all wines of high quality (awarded either a gold or double gold medal) are positioned in the middle of the score plot (Figure 2a), indicating volatile profiles without any extreme concentration levels. Along the first PC, wines on the left hand side of the score plot (W16, W12, W14) show higher levels in even-numbered ethyl esters with 6 to 16 C-atoms (C35, C54, C61, C63, C64) and an unidentified terpene (C32) compared to wines positioned on the right hand side of the score plot (e.g., W3, W13, W21, W25, W26). These latter wines show higher concentration levels in 35 volatiles, including various linear and branched aliphatic alcohols (C3, C7, C12, C13, C16, C34), phenylethanol (C50), acetic (C5) and 3-methylbutanoic acid (C19), butyl and acetyl esters (C6, C15, C26, C45, C56, C57), odd-numbered and branched ethyl esters (C10, C14, C18, C22, C23, C27, C47, C53, C62), together with limonene (C39), barrel-derived compounds such as oak lactone, furfural, difurfuryl ether (C20, C28−C30, C58−C59), aldehydes (C2, C43, C49), and mesifuran (2,5-dimethyl-4-methoxy-3(2H)-furanone C44). It seems that at least two underlying phenomena contribute to this separation:

(i) some of these compounds are related to ageing and/or oxidation reactions [37,38,39], e.g., ethyl-3-methyl butanoate (C23), diethyl succinate (C53), acetic acid (C5), and phenylethanol (C50) were reported to increase with increasing wine age, while various acetates, such as isoamyl acetate (3-methylbutyl acetate C25) decrease over time.

(ii) wines were also separated by the presence of barrel-derived compounds, such as furfural (C20), and oak lactone (C58, C59). Depending on the type and how much new oak barrels were used in the production of the wines, the concentration in these volatiles can vary significantly [40]. This is further substantiated by significant correlations to the oak aroma descriptor for (*Z*)- and (*E*)-oak lactone (C58, C59), and butyrolactone (C30) (Table 2).

Along the second dimension PC 2, explaining an additional 10% of the total variance, wines are separated by their varying levels in 18 volatiles—positively correlated are hexanoic and octanoic acid (C33, C51), linear aliphatic alcohols (C3, C24, C34), ethyl hexanoate (C41), furfural (C20), and SO_2_ (C1), while various ethyl esters (C6, C17, C21-C23, C53, C64), *p*-cymene (C38) and 4-ethylphenol (C52) are negatively correlated to PC 2. Besides a separation due to different wine age, expressed by the various acids, alcohols and esters, another phenomenon is apparent—whether the wine was affected by *Brettanomyces bruxellensis*, a spoilage yeast that is able to produce potent aroma compounds such as 4-ethylphenol, leading to typical “Brett” character, also described as barnyard, horse sweat, and leather, depending on the concentration levels and ratios of the involved compounds [41,42]. The levels of 4-ethylphenol measured in the wines were high enough for the trained panel to quantify, leading to a significant correlation between Brett aroma and 4-ethylphenol concentrations (Table 2). Another significant correlation between the sensory descriptor “Brett” was found for ethyl hexanoate (Table 2). Although not a major contributor to the typical Brett characters, ethyl hexanoate was reported to be produced by various Brettanomyces strains in wine [43].

Correlating the volatile compounds to the sensory attributes led to several significant relationships (Table 2): The two branched esters ethyl-2 and ethyl-3 methyl butanoate correlated significantly to overall aroma. The volatiles acetic acid (C5), ethyl acetate (C6), the branched alcohols C7, C12, and C13, the esters C15, C10, and C23, as well as 2-phenylethyl alcohol (C50), 2-phenylethyl acetate (C57), and phenylacetaldehyde (C43) correlated all positively to alcohol aroma. For canned vegetable aroma, two of three ethyl esters—ethyl pentanoate (C27) and ethyl heptanoate (C47)—showed a negative correlation (Table 2), indicating the absence of these compounds in wines that show high levels of canned vegetal aroma. On the other hand, ethyl-2-hexenoate (C42) was positively correlated to that sensory attribute (Table 2). Similarly for fresh green aroma, which showed a positive correlation to ethyl-2-hexenoate (C42), and negative correlations to the ethyl esters ethyl-9-decenoate (C60) and ethyl heptanoate (C47), as well as to limonene (C39) (Table 2). In the past, masking effects have been shown for fruity and green-vegetal attributes and compounds that are associated with these descriptors, such as β-damascenone and 2-methoxy-3-(2-methylpropyl)pyrazine (MIBP) [44,45].

Dark fruit aroma showed significant positive correlations to the ethyl and acetyl esters C9, C11, C27, C47, and C53 (Table 2). For red fruit aroma ethyl decanoate (C61) contributed positively while for 4-ethyl phenol (C52) a negative correlation was found. All these correlations are in agreement to previous studies that found that various linear and branched ethyl esters contribute to red and black berry aroma [39,44,46], and that high levels of 4-ethylphenol have a masking effect on fruit aroma perception [47].

For sweet aroma, described by the panel as *honey*, *caramel*, and *chocolate*, acetaldehyde (C5), various ethyl and acetyl esters (C6, C10, C27), as well as butyrolactone (C30) and acetoin (C9) all correlated positively, while a negative correlation between ethyl hexanoate (C41) and sweet aroma was found (Table 2). Similarly for spicy aroma, for which ground clove, cinnamon, nutmeg and ginger were used as reference standard in the DA: besides various linear and branched esters (C6, C9, C10, C14, C27, C56), furfural (C20), acetaldehyde (C2), phenyl acetaldehyde (C43), mesifuran (C44) and (E)-oak lactone (C59) all showed a positive correlation to spicy aroma. Again, ethyl hexanoate (C41) correlated negatively with spicy aroma (Table 2).

**Table 2:** Significant correlations (Pearson’s product-moment correlation coefficient r with df = 25, *p* < 0.05) between the volatile compounds and attributes from the DA.

Code	Overall Aroma	Alcohol	Brett	Canned Veggie	Fresh Green	Dark Fruit	Red Fruit	Dried Fruit	Sweet Aroma	Spice	Chemical	Earthy	Smoky	Soy Sauce	Sulfur	Oak	Astringent
**C2**									0.39	0.46							
**C5**		0.42															
**C6**		0.58							0.48	0.47							
**C7**		0.52															
**C8**																	
**C9**						0.41			0.62	0.4					−0.49		
**C10**		0.49						0.49	0.44	0.6							
**C11**						0.46		0.51			−0.52				−0.39		
**C12**		0.51															
**C13**		0.47															
**C14**										0.39							0.6
**C15**		0.59															
**C16**								0.48									
**C18**											−0.41						
**C20**										0.45							
**C22**	0.42										0.51						0.61
**C23**	0.38	0.45									0.5						0.56
**C24**																	−0.49
**C27**				−0.40		0.59		0.62	0.44	0.46					−0.41		
**C28**													0.48				
**C30**								0.43	0.41				0.41			0.60	
**C35**									−0.58								−0.51
**C39**					−0.41												
**C40**														0.52			
**C41**			0.47							−0.44							
**C42**				0.44	0.46												
**C43**		0.43								0.4				0.42			
**C44**										0.45							
**C45**														0.4			
**C47**				−0.39	−0.75	0.55		0.49									
**C49**																	0.61
**C50**		0.47					−0.5										
**C52**			0.71									0.39	0.46				
**C53**						0.45		0.44									0.49
**C54**														−0.55			
**C55**														0.44			
**C56**										0.41	0.42						
**C57**		0.57															
**C58**																0.47	
**C59**								0.49		0.45				0.45		0.63	
**C60**					−0.39												
**C61**							0.47							−0.61			
**C64**														−0.48			

Dried fruit aroma correlated positively to known ageing compounds such as diethyl succinate (C53), to oak-derived compounds such as oak lactone (C59) and butyrolactone (C30), to 2,3-butanediol (C16), and to various esters (C47, C27, C11, C10) (Table 2).

For chemical aroma, some higher esters showed a positive correlation (C56, C23, C22), while ethyl lactate (C18), and propyl acetate (C11) were significantly negatively correlated with the perceived chemical aroma impression.

4-Ethylphenol (4-EP, C52) played also a significant role in the perception of earthy aroma and smoky aroma (Table 2). For the latter aroma attribute, butyrolactone (C30), and difurfuryl ether (C28) correlated significantly as well (Table 2).

The impression of soy sauce was positively correlated to E-oak lactone (C59), octyl acetate (C55), isoamyl lactate (C45), phenylacetaldehyde (C43), and eucalyptol (C40). Negative correlations were found for higher ethyl esters with 8, 10 or 16 C-atoms (C54, C61, C64) (Table 2).

Sulfur aroma, as described by the panel as “*burnt rubber or rotten egg*”, showed mostly negative correlations to short-chain esters (C27, C11, C9) (Table 2), however, the most likely responsible volatile compounds for these aromas are low molecular sulfur compounds (e.g., sulfides and thiols) [48], which were not detected by the used HS-SPME-GC-MS method.

Although astringency is a mouthfeel sensation, elucidated by non-volatile polyphenols, some significant correlations to some volatile compounds were found (Table 2): Positive relationships were found for diethyl succinate (C53), nonanal (C49), and the branched ethyl esters C14, C22, and C23. San Juan *et al.* [47] reported that more expensive wines show higher concentrations of wood-related compounds and branched ethyl esters, but did not assess the astringency of their wines. Negative correlations to astringency were found for ethyl hexanoate (C41) and 1-hexanol (C24), similarly to [49]. The significant correlations between astringent mouthfeel and certain volatile compounds are strictly mathematical; in order to determine if there is a causal relationship between these parameters, this aspect has to be studied in future work.

All five quality indicators—judgment points, expert scores, bottle price, vintage, region—showed significant correlations to individual volatile compounds, which are summarized in Table 3.

Of all volatiles, only one single compound correlated significantly to the awarded points—limonene correlated negatively to awarded points (Table 3). For the wine expert scores, all significant correlations were negative; with increased levels of 1-butanol (C8), ethyl-2-methyl butanoate (C22), ethyl-3-methyl butanoate (C23), eucalyptol (C40), or 4-ethyl phenol (C52) wine experts scored the wines lower in quality (Table 3). This is in agreement with the correlations to the sensory attributes—experts agree more on low quality indicators, such as the presence of microbial spoilage (e.g., *Brettanomyces bruxellensis*) [31] or vegetal-green aromas [25]. Eucalyptol has been described as a major contributor to mint-like aromas [44].

Retail bottle price correlated positively (Table 3) to linear and branched ethyl esters (C6, C10, C14, C17, C55), as well as 2-methylpropyl acetate (C15), compounds that were found in higher concentrations in more expensive red wines in a previous study [47]. In the same study, wood-related compounds such as difurfuryl ether and oak lactone were present at higher levels in more expensive wines, an observation that is confirmed by our findings (Table 3): wood-derived compounds C28, C29, C58, and C59 all correlated positively to bottle price. Additional positive correlations between price and concentration levels were found for 4-ethyl phenol (C52), phenylacetaldehyde (C43), acetic acid (C5), p-cymene (C38), and nonanal (C49), similar to reports by San Juan *et al.* [47] for the former two compounds, while the latter three were reported to increase with storage temperature [23,50].

**Table 3:** Volatile compounds that showed significant correlations to the five quality proxies (Pearson’s product correlation coefficient r with df = 25, *p* < 0.05).

Code	Points	Expert	Price	Vintage	Regions
**C5**			0.42		
**C6**			0.62		
**C8**		−0.56			0.49
**C9**					0.51
**C10**			0.46		
**C14**			0.48	−0.66	
**C15**			0.41		
**C17**			0.49	−0.53	
**C20**					0.4
**C22**		−0.43		−0.86	
**C23**		−0.43		−0.81	0.41
**C24**				0.4	
**C27**				−0.42	
**C28**			0.45		
**C29**			0.49		
**C36**				0.38	
**C38**			0.39		−0.49
**C39**	−0.4				
**C40**		−0.47		−0.41	
**C41**				0.4	
**C43**			0.57		
**C48**				−0.44	
**C49**			0.49		
**C50**				−0.45	
**C52**		−0.47	0.77		
**C53**			0.53		
**C56**				−0.42	0.49
**C58**			0.72		
**C59**			0.42		
**C62**				−0.49	
**C64**					−0.38

The linear and branched ethyl and acetyl esters are known to contribute to the fresh, fruity and floral aromas in red wines (e.g., [51]), hence a significant negative correlation to vintage was observed for volatiles C14, C17, C22, C23, C27, C48, C56, and C62 (Table 3). A negative correlation to vintage was also found for phenylethanol (C50), similar to the report of higher levels of this compound in young red wines from Australia [51]. The fate of eucalyptol during wine storage is not fully understood, one study [52] reports that eucalyptol levels in model wine remain unchanged after two years under wine-like conditions, but the same study showed that wines from the same vineyard had lower eucalyptol levels for older vintages (up to 10 years old). This latter trend is suggested by our findings of a negative correlation to wine age for eucalyptol (C40). Three C6 compounds, namely 1-hexanol (C24), ethyl hexanoate (C35) and hexyl acetate (C36) all show a positive correlation with vintage (Table 3).

For seven volatiles, namely, 1-butanol (C8), acetoin (C9), furfural (C20), ethyl-3-methyl butanoate (C23), p-cymene (C38), isopentyl hexanoate (C56), and ethyl hexadecanoate (C64), significant regional differences were found (Table 3 and Table 4). However, ester content is heavily influenced during winemaking by the starting grape material (e.g., sugar levels, nitrogen content) and yeast strains [47,53]. Furfural is an aging-related compound [47], while p-cymene was reported after heated acid hydrolysis of grape-derived precursors [50]. It seems that these correlations are more a result of the different winemaking regimes exercised by the different wineries in the different regions. Only if all other parameters (winemaking, grape-growing, storage, *etc.*) are properly controlled could differences in volatile composition be attributed to different geographical regions.

**Table 4:** Mean concentrations in seven volatile compounds differed significantly among the nine wine regions in California, USA. Mean concentrations in the same column sharing a common lowercase letter are not significantly different from each other (*p* < 0.05). Mean values are calculated from two to four different wines per regions and three bottle replicates for each wine.

Region	Region Code	C8 (μg/L)	C9 (μg/L)	C20 (μg/L)	C23 (μg/L)	C38 (μg/L)	C56 (μg/L)	C64 (μg/L)
North Coast	A	835.3 b	255.1 abc	76.8 b	12.2 bc	1.66 a	926.9 cd	381.6 ab
Sonoma County	B	1113.0 ab	n.d. b	157.2 b	12.2 bc	1.26 ab	1659.0 bc	421.1 a
Napa County	C	1268.0 ab	85.7 b	194.4 b	17.7 abc	1.68 a	1956.0 bc	133.9 c
Greater Bay area	D	1372.0 a	294.8 ab	294.0 ab	20.6 ab	0.94 b	2256.0 ab	133.8 c
North Central Coast	E	1013.0 ab	310.2 b	110.1 b	5.9 c	1.07 ab	n.d. d	288.7 abc
South Central Coast	F	1243.0 ab	407.9 b	189.4 b	10.1 c	0.97 b	1905.0 bc	215.9 bc
South Coast	G	1551.0 a	728.7 ab	367.7 ab	17.1 abc	0.83 b	2086.0 ab	133.8 c
Sierra Foothills	H	1380.0 a	408.5 b	163.8 b	26.0 a	1.16 ab	2457.0 ab	262.3 abc
Lodi/Woodbridge	I	1429.0 a	957.5 a	645.2 a	24.6 a	0.85 b	3301.0 a	116.3 c

### 2.4. Elemental Profiling

**Figure 3:**
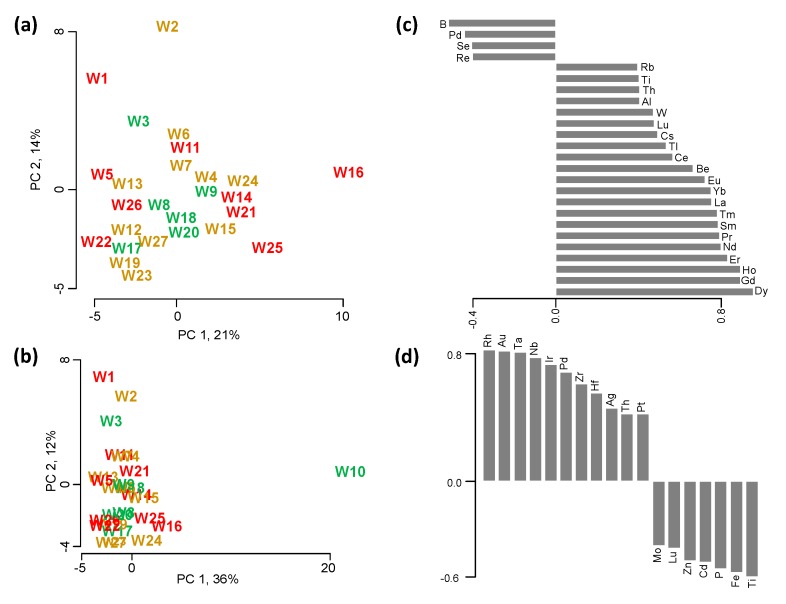
PCA of the elements that differed significantly among the 27 wines. (**a**) Score plot showing the elemental space of the wines, excluding wine W10. (**b**) Score plot showing the elemental space of all the wines, including wine W10. Wines are color-coded according to their assigned quality categories based on the wine judgment—red for wines low in quality, (*i.e*., that were awarded no medal), dark yellow for medium quality (*i.e*., either a bronze or silver medal), and green for high quality (*i.e*., a gold or double gold medal). Barplots of elements that contributed significantly (*p* < 0.05) to the separation of all wines but W10 (**c**) along the first principal component (PC 1), and (**d**) along the second principal component (PC 2).

A total of 54 elements (Table 8) in the mass range from 9–232 *m/z* were detected in the 27 wines using the described inductively-coupled plasma-mass spectrometry (ICP-MS) method. An additional six elements (Ca, K, Mg, Na, Rb, Sr) were measured with the described microwave-plasma-atomic emission spectrometry (MP-AES) method due to their high concentration levels in the wines (Table 9). Of these 60 elements, all differed significantly among the wines (*p* < 0.05) in the ANOVA, and were used in the PCA to obtain the elemental space of the studied wines, shown in Figure 3. Applying the Kaiser criterion and the scree test, the first two principal components (PCs) were kept, explaining 48% of the total variance. One wine (W10) showed very high concentrations of various rare earth elements (REEs), leading to a strong separation in the PCA between W10 and all other wines (Figure 3b). Therefore, another PCA without W10 was conducted, leading to the samples separation shown in Figure 3a. Excluding W10, the PCA explained 35% of the total variance in the first two dimensions, which were the dimensions retained due to the Kaiser criterion and scree test. In Figure 3c,d, all elements that correlated significantly along PC1 or PC 2 are displayed (*p* < 0.05). Along the first principal component (PC 1), wines are separated based on their levels in the REEs, Be, Tl, Cs, W, Al, Th, Ti and Rb on the right hand side of the plot *vs.* their concentration in B, Pd, Se and Re on the left hand side. Along the second dimension (PC 2), wines are separated based on their levels in Ti, Fe, P, Cd, Zn, Lu and Mo (bottom side of plot) vs. their content in Rh, Au, Ta, Nb, Ir, Pd, Zr, Hf, Ag, Th, and Pt (top side of plot). Although REEs were reported to be so called “natural elements” [54], present in the soil and taken up by the plant from the soil, several studies have shown that the REE content in wine can be dramatically increased by winemaking practices, such as filtration through silica, cellulose and bed filters [55], and clarification with bentonite [55,56,57], as well as during storage [55]. Based on these reports, we believe that wines W10 and to a lesser extent W16 were either filtered and/or clarified, leading to the dramatic increase in rare earth elements. Many elements can undergo changes in concentration during winegrowing and winemaking, including Rb, Ti, Al, W, Tl, and Be, as summarized in [21], while the same elements as well as Se, Cs, and the REEs have been applied in geographical classifications of wines all around the world [7,8,9,10,11,12,13,14,15,16,17,18,19,20]. It seems that the separation among the 27 wines is the combined “fingerprint” of geographical origin, viticulture, enology and storage conditions.

In a second step, elemental content of the wines were correlated to the various wine quality proxies to study potential elemental markers for wine quality. For the points awarded in the wine competition, only Hf showed a significant, negative correlation (*r*(25) = −0.41, *p* < 0.05). Bentonite, used in grape must clarification was reported as one source of Hf, which increased from below detection limit (<0.75 μg/L) to 1.5 μg/L [56].

For the expert scores, three elements all correlated positively, namely, the lighter rare earth element Eu (*r*(25) = 0.41, *p* < 0.05), Ba (*r*(25) = 0.44, *p* < 0.05), and Ga (*r*(25) = 0.41, *p* < 0.05).

No correlation to vintage was found for any of the detected elements, most likely due to the fact that there are no known universal elemental changes in wines over time, but rather elemental changes are depending on the individual elemental fingerprint.

Selenium (*r*(25) = −0.41, *p* < 0.05) and Cr (*r*(25) = −0.39, *p* < 0.05) both correlated negatively to retail price. While Cr was reported to be introduced into wine through the use of stainless steel equipment, Se was included in the classification of wines from different regions in New Zealand [14], Germany [11], South Africa [16], Australia [7], and Canada [9]. However, the correlation of these elements to retail price is most likely not causal.

Finally, six elements showed significant differences among the nine wine regions, thus showing a significant correlation to region, namely, Ba (*r*(25) = −0.62, *p* < 0.05), Be (*r*(25) = −0.52, *p* < 0.05), Ca (*r*(25) = 0.46, *p* < 0.05), Eu (*r*(25) = −0.43, *p* < 0.05), Ga (*r*(25) = −0.61, *p* < 0.05), and Pb (*r*(25) = 0.40, *p* < 0.05). Table 5 summarizes the regional differences in these six elements. Highest Ba levels were found for the wines from the North Coast region, while in the more southern coastal regions (E–G), and in the Sierra Foothills and Lodi/Woodbridge, the Ba concentrations were the lowest. For Ca, the lowest levels were found in Napa County while highest levels were found in the wines from the North Central Coast. Both Ba and Ca elements have been used in studies for the determination of geographical origin [7,8,9,10,11,12,13,14,15,16,17,18,19,20]. Calcium is present in the mg/L range in wine, and moderate wine consumption can be considered an important nutritional source for this element. Its source in wines can be both endo**-** and exogenous [58] Ca is an important element for the regulation of yeast metabolism during fermentation, it can be added as its salt form either as calcium carbonate or calcium sulfate to regulate the acidity of grape must, but is also present in vineyard soil [59], partly also due to the use of Ca-containing agrochemicals [58].

In contrast, Ba—present in wines between 0.01 and 0.48 mg/L [58]—was shown to differ in closely located vineyards, and was not significantly affected by winemaking [21]; thus, Ba differences among the regions could be the result of geographical differences.

Significantly higher Be levels compared to all other regions were found in the wines from the North Coast; Be was used in the classification of Canadian [8], and German wines [12], and together with Eu and Ga was not affected by winemaking in different wineries, but only due to vineyard location [21]. Both Eu and Ga ranked similarly across the different regions, except for Napa County, where Eu was significantly lower compared to the other regions, and Ga was significantly higher. It appears that some of these elemental differences could be related to the different geographical origins, however, for validating that these correlations could indeed be causal further work is needed.

**Table 5:** Mean concentrations of six elements show significant concentration differences among the nine wine regions in California, USA. Mean concentrations in the same column sharing a common lowercase letter are not significantly different (*p* < 0.05). Mean values are calculated from two to four different wines per regions and two bottle replicates for each wine.

Region	Region Code	Ba(μg/L)	Be (μg/L)	Ca (μg/L)	Eu (μg/L)	Ga(μg/L)	Pb (μg/L)
North Coast	A	518.1 a	0.4437 a	49575 ab	0.0507 a	28.45 a	3.841 ab
Sonoma County	B	358.6 abc	0.2087 ab	51803 ab	0.0210 ab	17.84 ab	1.960 b
Napa County	C	489.9 ab	0.1498 b	46509 b	0.0187 b	27.14 a	3.365 ab
Greater Bay area	D	343.1 abc	0.1177 b	53455 ab	0.0257 ab	17.97 ab	4.294 ab
North Central Coast	E	221.4 c	0.2690 ab	72253 a	0.0155 b	11.15 b	2.473 ab
South Central Coast	F	204.0 c	0.0753 b	59701 ab	0.0098 b	10.53 b	3.680 ab
South Coast	G	284.5 bc	0.1083 b	65295 ab	0.0153 b	14.97 b	6.798 a
Sierra Foothills	H	205.3 c	0.0700 b	61273 ab	0.0148 b	10.39 b	5.245 ab
Lodi/Woodbridge	I	243.2 c	0.1115 b	69804 ab	0.0160 b	13.22 b	5.183 ab

Lastly, lead levels differed across the regions, with highest levels in wines from the South Coast, and lowest in wines from Sonoma County—a more than three-fold difference. Lead is the only element in this group with a regulated maximum concentration limit in wine—150 μg/L in wines harvested in 2007 or later [60]. The origin of Pb in wine is due to environmental and wine production-related factors: first, Pb is present in soils, the atmosphere and the environment due to the prior use of leaded gasoline, but also industrial operations (e.g., mining and smelting) nearby [61], thus contributing about a third of the total lead content in finished wine, according to Almeida & Vasconcelos [62]. The same authors report that the majority of lead is introduced into wine during enological processes, more than tripling its initial lead content (4.1 μg/L) to 13.1 μg/L in finished red table wine. The use of lead as a welding alloy and in small fittings on tubes and containers were identified as the major sources. Based on this work we speculate that the significant higher Pb levels in wines from the South Coast may be the result of older winery equipment.

## 3. Experimental Section

### 3.1. Wine Samples

All wines used in this study are described in [25]. In summary, 27 different commercial Californian Cabernet Sauvignon wines (vintages 2001–2011; retail prices $9.99–$70) were selected based on their performance in the 2012 California State Fair Wine Competition (Table 6). The selected 27 wines were classified into 3 quality categories, based on their performance in the competition, with about a third of these wines (7 out of 27) deemed high in quality (*i.e*., awarded either a Gold or a Double Gold medal), 11 wines considered to be of medium quality (*i.e*., awarded either a Silver or a Bronze medal), and the remaining nine wines not receiving any medals, thus, were assigned to the low quality group. Two cases (= 24 bottles) of each wine were obtained, and used for all analyses. Wines were stored upright in the dark at 15 °C until use. All analyses were conducted within six months to each other to ensure comparability of the obtained results.

**Table 6:** Information about the 27 wines included in this study, including the various quality indicators—points awarded during the wine competition, assigned quality category, region, vintage, retail price, and wine expert ratings determined in this study.

Code	Vintage	Region ^a^	GDD ^b^	Points	Quality Category ^c^	EtOH (v/v %)	Closure	Retail Price	Expert Ratings
W1	2008	G	5621	82	low	14.3	synthetic	$26.95	49
W2	2009	B	3606	89	medium	14.9	natural	$39.00	70
W3	2009	I	4015	95	high	14.4	natural	$21.00	73
W4	2008	G	5621	90	medium	14.7	natural	$34.00	65
W5	2006	H	3612	83	low	13.9	natural	$15.00	51
W6	2009	C	3649	90	medium	14.5	natural	$55.00	97
W7	2010	H	3612	86	medium	14.6	natural	$25.00	95
W8	2008	C	3649	98	high	14.8	natural	$47.00	101
W9	2009	D	3786	94	high	14.5	natural	$25.00	94
W10	2009	A	3380	94	high	13.5	natural	$9.99	100
W11	2007	A	3380	82	low	14.2	natural	$38.00	68
W12	2009	F	3645	89	medium	13.5	screw cap	$15.00	88
W13	2007	D	3786	88	medium	14.8	natural	$34.00	66
W14	2008	B	3606	84	low	14.1	natural	$45.00	65
W15	2009	I	4015	89	medium	14.9	natural	$24.99	65
W16	2011	E	2919	82	low	13.5	synthetic	$10.00	77
W17	2009	F	3645	95	high	14.7	natural	$19.99	93
W18	2007	G	5621	98	high	14.5	natural	$70.00	66
W19	2010	F	3645	87	medium	13.5	screw cap	$22.00	80
W20	2010	B	3606	94	high	14.5	natural	$19.99	91
W21	2007	H	3612	83	low	13.7	natural	$29.00	95
W22	2010	F	3645	83	low	13.8	natural	$13.00	81
W23	2010	E	2919	89	medium	14.5	natural	$14.00	82
W24	2009	A	3380	88	medium	14.4	natural	$28.00	80
W25	2008	D	3786	82	low	14.7	natural	$32.00	59
W26	2009	C	3649	83	low	14.6	natural	$59.00	66
W27	2001	H	3612	92	medium	13.5	natural	$45.00	51

^a^ regions as defined in the California State Fair Wine Competition: A—North Coast; B—Sonoma County, C—Napa County, D—Greater Bay Area, E—North Central Coast, F—South Central Coast, G—South Coast, H—Sierra Foothills, I—Lodi/Woodbridge Grape Commission. ^b^ growing degree days as defined as “… a day on which the mean daily temperature is one degree above the base temperature-minimum temperature required for growth of a particular crop.” For grape, the base temperature is 50 °F/10 °C; data is given for the annual average and was extracted from the Western Regional Climate Center [63] for selected weather stations in each wine region (Lakeport for region A, Sonoma for region B, St. Helena for region C, Livermore for region D, Carmel Valley for region E, Paso Robles for region F, Hemet for region G, Placerville for region H, Lodi for region I). ^c^ quality categories were assigned by the authors based on the medals awarded in the wine competition. Wines were assigned to the low quality category when they did not receive any medals (<85 points), wines with bronze or silver medals were assigned to the medium quality category (85–93 points), and wines with either a gold or double gold medal were assigned to the high quality category (>94 points).

### 3.2. Chemicals

For the reference standards in the descriptive analysis (DA), food materials were used, as described in detail in [25]. Model wine (12% aqueous ethanol (v/v) (200 proof, GoldShield, Hayward, CA, USA), 5 g/L potassium bitartrate (Fisher Scientific, Pittsburgh, PA, USA), pH 3.3 adjusted with hydrochloric acid (Fisher Scientific) [64]), was used for the standard measurements in the volatile profiling.

Ultrapure concentrated nitric acid was obtained from Fisher Scientific and JT Baker (Center Valley, PA, USA). Ultrapure water (18 MΩ·cm, EMD Millipore Bellerica, MA, USA) and 200 proof ethanol (GoldShield) were used for the elemental calibration standards. Multielement calibration standards and the internal standard mix for the ICP-MS analyses, and Rb single-element standard for the MP-AES analyses were purchased from SPEX CertiPrep (Metuchen, NJ, USA). Other single-element calibration standards for MP-AES were obtained from VHG labs (Ca, K, Mg, Na; Manchester, NH, USA). The ionization buffer solution (100,000 mg/L Cs) was from Agilent Technologies (Santa Clara, CA, USA). All volatile compounds except those described below were purchased from Sigma-Aldrich (St. Louis, MO, USA; purity > 90%), as were acetaldehyde (natural 50% solution in ethanol), and sodium chloride. Ethyl-2-methylbutyrate (SAFISIS, Soustons, France), linalool (Alfa Aesar, Ward Hill, MA, USA), acetic acid (EMD, Merck, Darmstadt, Germany), 2-methylbutanoic acid (TCI America, Portland, OR, USA), hexanoic acid (Thermo Fisher Scientific, Geel, Belgium), and propionic acid (MP Biomedicals, Solon, OH, USA), all with a purity of >90%, were purchased from their respective producers.

### 3.3. Volatile Profiling Method

The volatile profiles of the wines were assessed using an automated headspace Solid Phase Microextraction-Gas Chromatography-Mass Spectrometry (HS-SPME-GC-MS) method, similarly to the methods described in [23,65]. A 2 cm mixed-phase SPME fiber (50/30 μm DVB/Carboxen/PDMS; Supelco, St. Louis, MO, USA) was employed for extraction and concentration of the volatile compounds. Five milliliters of wine, 2.0 ± 0.1 g of sodium chloride (Fisher Scientific), and 10 μL 2-undecanone [] as internal standard (IS) were placed in amber HS glass crimp vials (20 mL, Agilent Technologies, Santa Clara, CA, USA), and closed with a magnetic crimp cap (Supelco). Volatiles were extracted at 40 °C for 30 min and 250 rpm agitation (5 min incubation prior to extraction with 500 rpm agitation) with an autosampler (MPS2; Gerstel US, Linthicum Heights, MD, USA), and thermally desorbed for 16 min at 270 °C in the hot inlet equipped with a narrow diameter SPME inlet liner (Supelco). Each wine was prepared and analyzed in triplicate. Six different wines were analyzed per day, with each sample coming from a separate bottle. Replicate samples were spread through the analysis within the analysis day to control for potential sample aging while sitting on the autosampler.

An Agilent 7890A GC with a 5972C MS was used to separate and detect the extracted compounds, using an HP-5msUI column (30 m × 0.25 mm × 1 μm; Agilent Technologies), and an oven program as follows: 30 °C for 2 min, ramped with 5 °C/min to 180 °C, followed by a 20 °C/min ramp to 280 °C, with a final hold for 15 min. Separation was achieved using a constant Helium carrier gas flow (99.99% purity; Airgas, Sacramento, CA, USA) of 1 mL/min in split mode (20:1). Compounds were detected in the MS (MS source 240 °C, MS quadrupole 150 °C, MS transfer line 280 °C) using electron impact ionization (EI), in simultaneous selected ion monitoring (SIM) and Scan mode, providing both untargeted and targeted profiling [65]. In scan mode the MS scanned between 35 and 350 amu with 2.9 scans/sec, while in each of the 37 consecutive SIM windows between two and seven ions were detected with 45 of 50 ms dwell time for each ion.

Detected compounds were analyzed and areas were integrated in MSD Chemstation (version E.02.02, Agilent Technologies). Compounds were identified by matching their linear retention indices (RIs), calculated as described in [66] and mass spectra to pure standards, if available, and to mass spectral libraries (NIST/EPA/NIH Mass Spectral Library NIST 05). Identified compounds are listed in Table 7. Relative compound concentrations were calculated assuming a response factor of 1 between the compound and the internal standard (IS).

**Table 7:** Volatile compounds detected and quantified in this study. Shown are the mode of detection, as well as the calculated linear retention indices (RI) measured on a HP-5 ms column, the concentration ranges (minimum and maximum) expressed as μg internal standard equivalents (ISE) per liter wine, and the Fisher’s least significant different (LSD) concentrations (FDR < 0.05).

Code	Compound	Detection ^a^	RI ^b^	Min (μg/L)	Max (μg/L)	LSD (μg/L)
C1	sulfur dioxide ^c^	*m/z* 64	510	n.d. ^d^	1005	156
C2	acetaldehyde	SIM	516	n.d. ^d^	5.70	0.820
C3	1-propanol	SIM	572	n.d. ^d^	5.33	0.713
C4	2,3-butandione	SIM	591	n.d. ^d^	22.6	0.653
C5	acetic acid	*m/z* 60	593	533	3720	726
C6	ethyl acetate	SIM	611	309	903	113
C7	2-methyl-1-propanol	SIM	621	17.2	55.7	6.37
C8	1-butanol	SIM	653	708	2055	308
C9	acetoin	SIM	695	n.d. ^d^	1427	214
C10	ethyl propanoate	SIM	700	3.13	8.57	0.628
C11	*n*-propyl acetate	SIM	703	0.470	2.00	0.166
C12	3-methyl-1-butanol	SIM	725	195	447	51.0
C13	2-methyl-1-butanol	SIM	729	75.40	241	25.0
C14	ethyl 2-methylpropanoate	SIM	753	3.77	32.1	1.52
C15	2-methylpropyl acetate	SIM	769	1.57	6.30	0.379
C16	2,3-butandiol	SIM	772	n.d. ^d^	1732	726
C17	ethyl butanoate	SIM	800	12.10	27.30	1.72
C18	ethyl lactate	*m/z* 45	813	2974	16158	2019
C19	3-methylbutanoic acid	*m/z* 60	830	n.d. ^d^	184	24.2
C20	furfural	*m/z* 95	832	n.d. ^d^	1079	45.7
C21	ethyl (E)-2-butenoate	SIM	843	333	1234	72.6
C22	ethyl 2-methylbutanoate	SIM	850	1.83	32.8	1.01
C23	ethyl 3-methylbutanoate	SIM	853	3.13	37.7	1.36
C24	1-hexanol	SIM	867	10.0	62.5	6.19
C25	3-methylbutyl acetate	SIM	875	14.4	49.7	3.40
C26	2-methylbutyl acetate	SIM	878	1.87	5.50	0.335
C27	ethyl pentanoate	SIM	899	254	1381	65.2
C28	difurfuryl Ether ^c^	*m/z* 81	901	n.d. ^d^	4485	294
C29	difurfuryl Ether ^c^	SIM	901	n.d. ^d^	824	54.6
C30	butyrolactone	SIM	911	n.d. ^d^	361	115
C31	methyl hexanoate	SIM	924	n.d. ^d^	592	226
C32	unidentified terpene ^c^	SIM	942	n.d. ^d^	133	14.3
C33	hexanoic Acid	*m/z* 60	966	75.4	344	70.1
C34	1-heptanol	SIM	968	n.d. ^d^	1861	295
C35	ethyl hexanoate	SIM	997	124	300	16.0
C36	n-hexyl acetate	SIM	1010	1.47	10.4	0.403
C37	2-ethoxy-2-(2-furyl)ethanol ^c^	SIM	1024	n.d. ^d^	347	13.7
C38	*p*-cymene	SIM	1032	0.530	2.13	0.372
C39	limonene	SIM	1037	n.d. ^d^	5.33	0.764
C40	eucalyptol	SIM	1041	n.d. ^d^	632	39.9
C41	ethyl hexanoate	*m/z* 97	1042	33.8	202	11.5
C42	ethyl 2-hexenoate ^c^	SIM	1042	n.d. ^d^	3.67	0.246
C43	phenylacetaldehyde	SIM	1050	0.97	2.57	0.579
C44	2,5-dimethyl-4-methoxy-3(2H)-furanone	SIM	1061	n.d. ^d^	1357	123
C45	isoamyl lactate ^c^	SIM	1070	1.03	4.17	0.662
C46	(*Z*)-linalool oxide	SIM	1079	n.d. ^d^	401	12.5
C47	ethyl heptanoate	SIM	1095	1.03	6.13	0.210
C48	linalyl acetate	SIM	1100	n.d. ^d^	854	68.8
C49	nonanal	SIM	1104	n.d. ^d^	852	257
C50	phenethyl alcohol	SIM	1121	44.6	206	42.84
C51	octanoic Acid	*m/z* 60	1159	n.d. ^d^	382	94.3
C52	4-ethylphenol	SIM	1167	n.d. ^d^	15117	3795
C53	diethyl succinate	SIM	1175	227	930	151
C54	ethyl octanoate	SIM	1194	303	855	40.0
C55	n-octyl acetate	SIM	1208	n.d. ^d^	1437	67.3
C56	isopentyl hexanoate ^c^	SIM	1250	n.d. ^d^	3944	458
C57	2-phenethyl acetate	SIM	1264	1.80	5.70	1.10
C58	(*Z*)-oaklactone	*m/z* 99	1300	130	2031	315
C59	(*E*)-oaklactone	SIM	1337	n.d. ^d^	4084	417
C60	ethyl 9-decenoate	SIM	1388	n.d. ^d^	3898	171
C61	ethyl decanoate	SIM	1394	26.2	157	14.3
C62	ethyl 3-methylbutyl succinate ^c^	SIM	1430	0.700	4.87	0.883
C63	ethyl dodecanoate	SIM	1594	0.570	6.80	0.736
C64	ethyl hexadecanoate	SIM	1994	75.3	706	121

^a^ Compound areas were extracted either from the SIM trace (SIM), or via the extracted ion from the scan trace (*m/z*); ^b^ RI were matched with RI libraries available on the internet [67,68,69]; ^c^ tentatively identified—no reference standard; ^d^ not detected.

### 3.4. Elemental Profiling

The elemental composition of the wines was measured with a 7700x Inductively-Coupled Plasma-Mass Spectrometer (ICP-MS) from Agilent Technologies, using the same conditions as described in [21]. In short, wines were diluted, in duplicate, 1:3 in 5% Nitric Acid (Fisher Scientific) in metal-free 50 mL plastic tubes (VWR, Radnor, PA, USA) prior to analysis. The IS mix was diluted 1:10 in 1% HNO_3_ and constantly fed into the sample stream before entering the spray chamber (quartz double wall, cooled to 2 °C). Sample was introduced into the spray chamber through a MicroMist nebulizer (Agilent). The plasma was operated at a RF power of 1550 W, a RF matching voltage of 1.8 V, a sampling depth of 10 mm, and an Argon (99.999% purity; Airgas) carrier gas flow of 1.05 L/min. Elements listed in Table 8 were monitored using the ORS3 system in no gas, helium (He flow 4.3 mL/min) and/or high energy helium (He flow 10 mL/min) gas mode, and calibrated between 0 and 500 μg/L with matrix-matched calibration standards (4% ethanol, 5% HNO_3_) to account for possible matrix effects on the plasma stability [55,70,71,72]. Detection limits were calculated from the measurement of 10 calibration blanks [73]. Data was analyzed in ICP-MS MassHunter software (version B.01.03; Agilent Technologies).

**Table 8:** Elements detected by ICP-MS, together with calibration parameters (correlation coefficient R, intercept d, slope k), detection modes, limits of detection (LOD), and detected concentration ranges (minimum and maximum).

Element		Mode ^a^	R	d	k (Blank)	LOD ^b^ (μg/L)	Min (μg/L)	Max (μg/L)
9	Be	ng	0.99999	0.00131	0.00001	0.0057	0.016	0.768
11	B	ng	0.99999	0.00090	0.00386	5.3657	5140.718	18367.781
27	Al	He	0.99999	0.00090	0.00159	0.0788	140.93	1001.583
28	Si	He	0.99964	0.00059	0.59768	13.6102	11802.427	48636.718
31	P	He	0.99981	0.00013	0.00111	2.6963	258706.776	571219.68
34	S	He	0.94825	0.00000	0.00913	327.0532	294596.986	803247.824
47	Ti	He	0.99990	0.00098	0.00016	0.4161	15.126	75.573
51	V	He	0.99990	0.03808	0.00101	0.0040	0.238	438.866
52	Cr	He	0.99990	0.04921	0.01665	0.0228	5.732	70.931
55	Mn	He	0.99990	0.02531	0.00175	0.0428	779.293	3281.82
56	Fe	He	0.99990	0.04142	0.04786	0.0521	389.695	2891.526
59	Co	He	0.99990	0.08494	0.00043	0.0020	1.323	11.049
60	Ni	He	0.99990	0.02387	0.00364	0.4025	8.704	101.587
63	Cu	He	0.99990	0.07832	0.02022	0.0158	11.332	350.268
66	Zn	He	0.99990	0.01436	0.03622	0.0368	283.243	1576.367
69	Ga	ng	0.99990	0.03555	0.00014	0.0006	3.311	43.356
75	As	He	0.99994	0.02277	0.00050	0.0024	0.462	34.446
77	Se	heHe	1.00000	0.00089	0.00001	0.0124	0.347	3.841
90	Zr	He	0.99991	0.01087	0.00221	0.0044	n.d.	2.033
93	Nb	He	0.99997	0.02358	0.00014	0.0018	0.014	0.207
95	Mo	ng	0.99999	0.00181	0.00006	0.0056	0.544	24.833
103	Rh	He	0.99998	0.04102	0.00001	0.0002	0.001	0.016
105	Pd	He	0.99992	0.00858	0.00071	0.0027	0.06	1.209
107	Ag	He	0.99997	0.01783	0.00004	0.0016	0.002	0.012
111	Cd	ng	0.99999	0.00110	0.00001	0.0030	0.085	3.759
118	Sn	ng	0.99995	0.00297	0.00020	0.0053	n.d.	2.808
121	Sb	He	0.99997	0.00636	0.00008	0.0045	0.041	3.564
125	Te	He	1.00000	0.00029	0.00000	0.0000	0.000	0.038
133	Cs	ng	0.99999	0.01079	0.00001	0.0002	1.470	68.223
137	Ba	ng	0.99996	0.00138	0.00004	0.0052	60.525	603.697
139	La	He	0.99997	0.02344	0.00001	0.0002	0.009	1.493
140	Ce	He	0.99997	0.02768	0.00002	0.0002	0.013	2.376
141	Pr	He	0.99998	0.02765	0.00000	0.0005	0.002	0.398
146	Nd	He	0.99994	0.00546	0.00000	0.0011	0.008	1.618
147	Sm	He	0.99995	0.00470	0.00000	0.3430	n.d.	0.343
153	Eu	He	0.99997	0.01777	0.00001	0.1070	n.d.	0.107
157	Gd	He	0.99995	0.00751	0.00000	0.0005	0.004	0.331
163	Dy	ng	0.99999	0.00391	0.00000	0.3290	n.d.	0.329
165	Ho	He	0.99997	0.04206	0.00001	0.0002	0.001	0.068
166	Er	He	0.99997	0.01437	0.00000	0.0003	0.004	0.205
169	Tm	He	1.00000	0.04558	0.00002	0.0360	n.d.	0.036
172	Yb	He	0.99996	0.01033	0.00000	0.0002	0.009	0.319
175	Lu	He	0.99906	0.03030	0.00803	0.0123	n.d.	0.097
178	Hf	He	0.99906	0.00260	0.00008	0.0056	0.073	0.854
181	Ta	He	1.00000	0.01683	0.00014	0.0009	0.046	0.705
182	W	He	0.99994	0.00439	0.00004	0.0018	0.061	9.561
185	Re	ng	0.99999	0.00396	0.00001	0.4770	n.d.	0.477
193	Ir	He	0.99997	0.01032	0.00000	0.0920	n.d.	0.092
195	Pt	He	0.99996	0.00381	0.00001	0.0210	n.d.	0.021
197	Au	He	0.99995	0.00646	0.00001	0.0007	0.024	0.357
205	Tl	He	0.99996	0.01102	0.00002	0.0006	0.110	1.216
208	Pb	He	0.99996	0.01455	0.00043	0.0031	1.422	9.331
232	Th	He	0.99995	0.01596	0.00006	0.1230	n.d.	0.123
238	U	He	1.00000	0.01652	0.00001	0.0002	0.004	1.086

^a^ detection mode: ng: no gas, He: helium mode (flow 4.3 mL/min), heHe: high energy helium mode (flow 10 mL/min); ^b^ limit of detection (LOD) determined with 10 calibration blank runs.

Higher concentration elements (>500 μg/L; Ca, K, Mg, Na, Rb, Sr) were determined with a 4200 Microwave Plasma-Atomic Emission Spectrometry (MP-AES; Agilent Technologies), using the same conditions as described in [74]. Briefly, wine samples were diluted 1:50 in 5% HNO_3_, and the ionization buffer, diluted to 2000 mg/L in 1% HNO_3_, was constantly mixed with the sample stream in a mixing tee before entering the spray chamber (baffled cyclonic held at room temperature; Agilent Technologies). A micromist nebulizer (Agilent Technologies) was used for sample transport. An external gas control module (EGCM) was employed to reduce carbon build-up in the torch by injecting air into the nitrogen plasma. Each wine sample was analyzed in triplicate, using the conditions listed in Table 9. A six-point calibration between 0 and 20 mg/L (*i.e*., equivalent to 0–1000 mg/L in the samples) was carried out in matrix-matched solutions (5% HNO3 and 0.2% ethanol) to account for a possible matrix effect by the presence of ethanol. Detection limits (LOD) were calculated from 10 calibration blanks [73]. Collected data was analyzed in MP Expert software (Agilent Technologies).

**Table 9:** Instrument settings for the measurement of higher concentrated elements, using MP-AES, together with calibration parameters, limits of detection (LOD), detected ranges, and Fisher’s least significant differences (LSD) (FDR < 0.05) for each monitored element.

Element	Sr	Rb	Mg	Ca	Na	K
Monitored Wavelength [nm]	407.771	780.027	279.553	396.847	589.592	769.897
EGCM setting	Low	Low	Med	High		
Pump rate [rpm]	10					
Read time [s]	5		2			
Calibration range [mg/L]	0–5	0–5	0–5	0–5	0–5	0–20
Correlation coefficient	0.9999	0.9997	0.9998	0.9999	0.9999	0.9999
LOD ^a^ [mg/L]	0.0018	0.0004	0.0012	0.0016	0.0007	0.0020
Min [mg/L]	0.298	0.702	52.643	31.013	5.357	677.555
Max [mg/L]	1.301	8.831	142.285	75.943	49.693	1620.74
LSD [mg/L]	0.0584	0.0775	0.9159	0.4584	0.4277	24.4880

^a^ determined from 10 calibration blank measurements.

### 3.5. Sensory Profiling and Expert Tasting

All wines were characterized with Descriptive Analysis (DA), a qualitative and quantitative sensory technique where a trained sensory panel provides a sensory profile [75]. All details with regards to the DA are given in [25], and are briefly summarized here: The panel consisted of 10 males and five females (average age 37 ± 17 years), who volunteered to participate in the study. They were recruited via emails from the UC Davis community, and included students, staff, and retirees. Each panelist gave oral consent prior for inclusion before they participated in the study. The study was evaluated and approved by the local institutional review board (UC Davis IRB; protocol 305379-2), and was conducted in accordance with the Declaration of Helsinki. Panelists underwent 6 one-hour training sessions over a period of two weeks where they were exposed to subsets of the 27 wines. During training, the panelists created, refined and agreed upon sensory attributes (21 aroma, 3 taste, 3 mouthfeel descriptors) that described the differences they perceived among the wines. For each sensory attribute, a corresponding reference standard was defined, and the panel had to blindly recognize these references at the end of the training, before they were able to proceed to the wine assessment. All wines were assessed in triplicate in individual tasting booths under red light and positive air pressure. During each session, the panelists were presented with 6–7 wines in black tasting glasses (25 mL each), each labeled with an individual three-digit random code. Samples were presented in a William-Latin square block design to control for carry-over effects [76]. Each attribute was rated on a computer screen on an unstructured line scale (0–9), anchored at both ends, using the FIZZ software (version 2.47B, Biosystèmes, Couternon, France).

All 27 wines were also assessed for their quality with a group of 28 wine professionals, *i.e.*, people that work in the wine and related industries in jobs where business decisions are based on the outcome of wine tastings. Details are given in [25], but are briefly summarized: Experts were presented with 30 glasses of wine (27 samples and 3 blind duplicates), labeled with random three-digit codes, and asked to score each wine on a 9-point category scale according to their liking of the wine. The left end of the scale was labeled with “Dislike extremely”, in the middle with “Neither like nor dislike”, and the right end of the scale was labeled with “Like extremely. In the past, studies [25,77] have shown that hedonic liking is directly correlated to perceived quality, independent of wine expertise. The obtained liking scores of the experts were averaged for each wine over the 28 experts, and used as a quality measurement (“expert rating”).

### 3.6. Data Analysis

Statistical significance was set for all data analyses at 5%. Sensory profiling data was analyzed by analysis of variance (ANOVA) for the main effects wine, sensory replicate, and judge, as well as for all two-way interactions. For significant wine-by-judge interactions, a pseudo-mixed model with the interaction sum of squares as error term was applied to test if the wine effect remained significant [78]. Volatile compound concentrations and elemental concentrations from ICP-MS and MP-AES were subjected to statistical analysis for significant sample and analytical replicate effects, using ANOVA. Post-hoc mean separation was carried out using Fisher’s least significant differences (LSDs), applying false-discovery-rate (FDR) adjustment for multiple comparisons.

Multivariate data interpretation was facilitated by principal component analysis (PCA) on the correlation matrix (data scaled to unit variance and zero mean) for each data set (sensory profile, volatiles, elements). Decision on how many principal components to keep was based on the Kaiser criterion and the scree test [79,80]. Simple correlations between individual components (*i.e.*, volatile compounds, elements, sensory descriptors) and the various quality measurements (wine score, expert rating, vintage, bottle price, region) were calculated as the Pearson product-moment correlation coefficient r. Data were prepared for analyses in Excel 2013 (Microsoft, Redmond, DC, USA). All data analyses were done in RStudio (version 0.98.978, Boston, MA, USA), with the basic R packages (version 3.1.0, R Core Team, Vienna, Austria) and the additional R packages FactoMineR [81,82], deducer [83] and agricolae [84].

## 4. Conclusions

The complexity of wine quality is best described by the many measures of wine quality—common proxies for wine quality include expert evaluation scores given by wine critics, wine experts or during wine judgments, as well as retail price, the year the wine was made (=vintage) and the geographical origin. For each of these proxies, analytical methods can be used to correlate the measured wine components to the quality proxies.

In this study, the sensory, volatile and elemental profiles of 27 Californian Cabernet Sauvignon wines were correlated to the quality proxies (i) points awarded during a wine competition, (ii) wine expert liking scores, (iii) retail bottle price, (iv) vintage, and (v) wine region. With the exception of points awarded during a wine competition and the wine expert scores, none of the quality proxies showed significant correlations (*p* < 0.05) to each other, indicating that they in fact cover different aspects of wine quality. For the sensory attributes from the descriptive analysis panel, both quality proxies as well as several volatile compounds showed significant correlations, pointing on one hand to the importance of wine flavor perception to overall wine quality as judged by wine experts, and on the other hand towards the importance of the volatile fraction to wine quality. However, as expected, no single compound or sensory descriptor is able to fully describe all aspects of wine quality. Lastly, the elemental profile was able to add an unexplored element to wine quality: regionality or regional typicity is considered a proxy for wine quality. Correlating the diverse elemental profiles of the studied wines to the various quality proxies, one thing becomes apparent: without full knowledge of each wine’s history, including viticultural and enological treatments, the elemental fingerprint can only serve as a pointer towards regionality. The results of this study can only serve as an initial look at quality correlations to instrumental parameters. It is therefore necessary to validate the reported findings on a more extensive sample set, both with regards to number of wines per region and wines per quality category.

## References

[1] Charters S., Pettigrew S. (2007). The dimensions of wine quality. Food Qual. Prefer..

[2] Verdú Jover A.J., Lloréns Montes F.J., Fuentes Fuentes M.D.M. (2004). Measuring perceptions of quality in food products: The case of red wine. Food Qual. Prefer..

[3] Thach L. (2008). How American Consumers Select Wine. Wine Bus. Mon..

[4] Austrian Wine Law. http://www.ris.bka.gv.at/GeltendeFassung.wxe?Abfrage=Bundesnormen&Gesetzesnummer=20006757.

[5] D’Alessandro S., Pecotich A. (2013). Evaluation of wine by expert and novice consumers in the presence of variations in quality, brand and country of origin cues. Food Qual. Prefer..

[6] Kelly S., Heaton K., Hoogewerff J. (2005). Tracing the geographical origin of food: The application of multi-element and multi-isotope analysis. Trends Food Sci. Technol..

[7] Martin A.E., Watling R.J., Lee G.S. (2012). The multi-element determination and regional discrimination of Australian wines. Food Chem..

[8] Greenough J.D., Longerich H.P., Jackson S.E. (1997). Element fingerprinting of Okanagan Valley wines using ICP-MS : Relationships between wine composition, vineyard and wine colour. Aust. J. Grape Wine Res..

[9] Taylor V.F., Longerich H.P., Greenough J.D. (2003). Multielement Analysis of Canadian Wines by Inductively Coupled Plasma Mass Spectrometry (ICP-MS) and Multivariate Statistics. J. Agric. Food Chem..

[10] Sperkova J., Suchanek M. (2005). Multivariate classification of wines from different Bohemian regions (Czech Republic). Food Chem..

[11] Castiñeira Gómez M.D.M., Feldmann I., Jakubowski N., Andersson J.T. (2004). Classification of German white wines with certified brand of origin by multielement quantitation and pattern recognition techniques. J. Agric. Food Chem..

[12] Thiel G., Geisler G., Blechschmidt I., Danzer K. (2004). Determination of trace elements in wines and classification according to their provenance. Anal. Bioanal. Chem..

[13] Marengo E., Aceto M. (2003). Statistical investigation of the differences in the distribution of metals in Nebbiolo-based wines. Food Chem..

[14] Angus N.S., O’Keeffe T.J., Stuart K.R., Miskelly G.M. (2006). Regional classification of New Zealand red wines using inductively-coupled plasma-mass spectrometry (ICP-MS ). Aust. J. Grape Wine Res..

[15] Coetzee P.P., Vanhaecke F. (2005). Classifying wine according to geographical origin via quadrupole-based ICP-mass spectrometry measurements of boron isotope ratios. Anal. Bioanal. Chem..

[16] Coetzee P.P., Steffens F.E., Eiselen R.J., Augustym O.P., Balcaen L., Vanhaecke F. (2005). Multi-element Analysis of South African Wines by ICP-MS and Their Classification According to Geographical Origin. J. Agric. Food Chem..

[17] Baxter M.J., Crews H.M., Dennis M.J., Goodall I., Anderson D. (1997). The determination of the authenticity of wine from its trace element composition. Food Chem..

[18] Iglesias M., Besalú E., Anticó E. (2007). Internal standardization—Atomic Spectrometry and geographical pattern recognition techniques for the multielement analysis and classification of Catalonian red wines. J. Agric. Food Chem..

[19] Pérez Trujillo J.P., Conde J.E., Pérez Pont M.L., Câmara J., Marques J.C. (2011). Content in metallic ions of wines from the Madeira and Azores archipelagos. Food Chem..

[20] Sen I., Tokatli F. (2013). Characterization and Classification of Turkish Wines Based on Elemental Composition. Am. J. Enol. Vitic..

[21] Hopfer H., Nelson J., Collins T.S., Heymann H., Ebeler S.E. (2015). The combined impact of vineyard origin and processing winery on the elemental profile of red wines. Food Chem..

[22] Hopfer H., Ebeler S.E., Heymann H. (2012). How Blending Affects the Sensory and Chemical Properties of Red Wine. Am. J. Enol. Vitic..

[23] Hopfer H., Buffon P.A., Ebeler S.E., Heymann H. (2013). The Combined Effects of Storage Temperature and Packaging on the Sensory, Chemical, and Physical Properties of a Cabernet Sauvignon Wine. J. Agric. Food Chem..

[24] Hopfer H., Ebeler S.E., Heymann H. (2012). The Combined Effects of Storage Temperature and Packaging Type on the Sensory and Chemical Properties of Chardonnay. J. Agric. Food Chem..

[25] Hopfer H., Heymann H. (2013). Judging wine quality: Do we need experts, consumers or trained panelists?. Food Qual. Prefer..

[26] Hodgson R.T. (2009). An Analysis of the Concordance Among 13 U.S. Wine Competitions. J. Wine Econ..

[27] Gawel R., Godden P.W. (2008). Evaluation of the consistency of wine quality assessments from expert wine tasters. Aust. J. Grape Wine Res..

[28] King E.S., Dunn R.L., Heymann H. (2013). The influence of alcohol on the sensory perception of red wines. Food Qual. Prefer..

[29] McRae J.M., Kennedy J.A. (2011). Wine and Grape Tannin Interactions with Salivary Proteins and Their Impact on Astringency: A Review of Current Research. Molecules.

[30] Harbertson J.F., Parpinello G.P., Heymann H., Downey M.O. (2012). Impact of exogenous tannin additions on wine chemistry and wine sensory character. Food Chem..

[31] Lattey K.A., Bramley B.R., Francis I.L. (2010). Consumer acceptability, sensory properties and expert quality judgements of Australian Cabernet Sauvignon and Shiraz wines. Aust. J. Grape Wine Res..

[32] Oelofse A., Pretorius I.S., du Toit M. (2008). Significance of Brettanomyces and Dekkera during Winemaking: A Synoptic Review. S. Afr. J. Enol. Vitic..

[33] Schmid F., Grbin P., Yap A., Jiranek V. (2011). Relative efficacy of high-pressure hot water and high-power ultrasonics for wine oak barrel sanitization. Am. J. Enol. Vitic..

[34] Henick-Kling T., Egli C., Licker J., Mitrakul C., Acree T.E. Brettanomyces in wine. Proceedings of the 5th International Symposium on Cool Climate Viticulture and Oenology.

[35] Goldner M.C., Zamora M.C., Lira P.D.L., Gianninoto H., Bandoni A. (2009). Effect of ethanol level in the perception of aroma attributes and the detection of volatile compounds in red wine. J. Sens. Stud..

[36] Gambuti A., Rinaldi A., Ugliano M., Moio L. (2013). Evolution of phenolic compounds and astringency during aging of red wine: Effect of oxygen exposure before and after bottling. J. Agric. Food Chem..

[37] Lee D.H., Kang B.S., Park H.J. (2011). Effect of oxygen on volatile and sensory characteristics of Cabernet Sauvignon during secondary shelf life. J. Agric. Food Chem..

[38] Bueno M., Culleré L., Cacho J., Ferreira V. (2010). Chemical and sensory characterization of oxidative behavior in different wines. Food Res. Int..

[39] Ferreira V., San Juan F., Jelen H. (2011). Flavor of Wine. Food Flavors.

[40] Pérez-Prieto L.J., López-Roca J.M., Martínez-Cutillas A., Pardo Mínguez F., Gómez-Plaza E. (2002). Maturing Wines in Oak Barrels. Effects of Origin, Volume, and Age of the Barrel on the Wine Volatile Composition. J. Agric. Food Chem..

[41] Suárez R., Suárez-Lepe J.A., Morata A., Calderón F. (2007). The production of ethylphenols in wine by yeasts of the genera Brettanomyces and Dekkera: A review. Food Chem..

[42] Joseph C.M.L., Gorton L.W., Ebeler S.E., Bisson L.F. (2013). Production of Volatile Compounds by Wine Strains of Brettanomyces bruxellensis Grown in the Presence of Different Precursor Substrates. Am. J. Enol. Vitic..

[43] Romano A., Perello M.C., de Revel G., Lonvaud-Funel A. (2008). Growth and volatile compound production by Brettanomyces/Dekkera bruxellensis in red wine. J. Appl. Microbiol..

[44] Robinson A.L., Adams D.O., Boss P.K., Heymann H., Solomon P.S., Trengove R.D. (2011). The relationship between sensory attributes and wine composition for Australian Cabernet Sauvignon wines. Aust. J. Grape Wine Res..

[45] Hein K., Ebeler S.E., Heymann H. (2009). Perception of fruity and vegetative aromas in red wine. J. Sens. Stud..

[46] Pineau B., Barbe J.C., van Leeuwen C., Dubourdieu D. (2009). Examples of perceptive interactions involved in specific “Red-” and “Black-berry” aromas in red wines. J. Agric. Food Chem..

[47] Juan F.S., Cacho J., Ferreira V., Escudero A. (2012). Aroma chemical composition of red wines from different price categories and its relationship to quality. J. Agric. Food Chem..

[48] Herszange J., Ebeler S.E. (2011). Analysis of volatile organic sulfur compounds in wine using Headspace Solid-Phase Microextraction Gas Chromatography with Sulfur Chemiluminescence Detection. Am. J. Enol. Vitic..

[49] Sáenz-Navajas M.P., Campo E., Fernández-Zurbano P., Valentin D., Ferreira V. (2010). An assessment of the effects of wine volatiles on the perception of taste and astringency in wine. Food Chem..

[50] Robinson A.L., Mueller M., Heymann H., Ebeler S.E., Boss P.K., Solomon P.S., Trengove R.D. (2010). Effect of Simulated Shipping Conditions on Sensory Attributes and Volatile Composition of Commercial White and Red Wines. Am. J. Enol. Vitic..

[51] Francis I.L., Newton J.L. (2005). Determining wine aroma from compositional data. Aust. J. Grape Wine Res..

[52] Capone D.L., van Leeuwen K., Taylor D.K., Jeffery D.W., Pardon K.H., Elsey G.M., Sefton M.A. (2011). Evolution and occurrence of 1,8-cineole (Eucalyptol) in Australian wine. J. Agric. Food Chem..

[53] Vianna E., Ebeler S.E. (2001). Monitoring ester formation in grape juice fermentations using solid phase microextraction coupled with gas chromatography-mass spectrometry. J. Agric. Food Chem..

[54] Volpe M.G., La Cara F., Volpe F., de Mattia A., Serino V., Petitto F., Zavalloni C., Limone F., Pellecchia R., de Prisco P.P. (2009). Heavy metal uptake in the enological food chain. Food Chem..

[55] Rossano E.C., Szilágyi Z., Malorni A., Pocsfalvi G. (2007). Influence of winemaking practices on the concentration of rare earth elements in white wines studied by inductively coupled plasma mass spectrometry. J. Agric. Food Chem..

[56] Castiñeira Gómez M.D.M., Brandt R., Jakubowski N., Andersson J.T. (2004). Changes of the metal composition in German white wines through the winemaking process. A study of 63 elements by inductively coupled plasma-mass spectrometry. J. Agric. Food Chem..

[57] Jakubowski N., Brandt E., Stuewer D., Eschnauer H.R., Görtges S. (1999). Analysis of wines by ICP-MS: Is the pattern of the rare earth elements a reliable fingerprint for the provenance ?. Fresenius. J. Anal. Chem..

[58] Pohl P. (2007). What do metals tell us about wine?. TrAC Trends Anal. Chem..

[59] Almeida C.M.R., Vasconcelos M.T.SD. (2003). Multielement composition of wines and their precursors including provenance soil and their potentialities as fingerprints of wine origin. J. Agric. Food Chem..

[60] Organisation de Vin (OIV) (2011). OIV-MA-C1-01: R2011 Maximum Acceptable Limits of Various Substances Contained in Wine.

[61] Tong S., Von Schirnding Y.E., Prapamontol T. (2000). Environmental lead exposure: A public health problem with global dimensions. Bull. World Health Organ..

[62] Almeida C.M.R., Vasconcelos M.T.S.D. (2003). Lead contamination in Portuguese red wines from the Douro region: from the vineyard to the final product. J. Agric. Food Chem..

[63] Western Regional Climate Center. http://www.wrcc.dri.edu.

[64] Harbertson J.F., Picciotto E.A., Adams D.O. (2003). Measurement of Polymeric Pigments in Grape Berry Extract sand Wines Using a Protein Precipitation Assay Combined with Bisulfite Bleaching. Am. J. Enol. Vitic..

[65] Hjelmeland A.K., King E.S., Ebeler S.E., Heymann H. (2013). Characterizing the chemical and sensory profiles of United States Cabernet Sauvignon wines and blends. Am. J. Enol. Vitic..

[66] IUPAC Goldbook: Retention Index. http://goldbook.iupac.org/R05360.html.

[67] Acree T., Arn H. Flavornet and human odor space. http://flavornet.org.

[68] Sayed A.M. The Pherobase: Database of Pheromones and Semiochemicals. http://www.pherobase.com.

[69] NIST Chemistry WebBook. http://webbook.nist.gov.

[70] Boorn A.W., Browner R.F. (1982). Effects of organic solvents in inductively coupled plasma atomic emission spectrometry. Anal. Chem..

[71] Dams R.F.J., Goossens J., Moens L. (1995). Spectral and non-spectral interferences in inductively coupled plasma mass-spectrometry. Mikrochim. Acta.

[72] Goossens J., Moens L., Dams R. (1994). Determination of lead by flow-injection inductively coupled plasma mass spectrometry comparing several calibration techniques. Anal. Chim. Acta.

[73] Thomsen V., Schatzlein D., Mercuro D. (2003). Limits of Detection in Spectroscopy. Spectroscopy.

[74] Nelson J., Hopfer H., Gilleland G., Cuthbertson D., Boulton R., Ebeler S.E. (2015). Elemental Profiling of Malbec Wines Made under Controlled Conditions by Microwave Plasma Atomic Emission Spectroscopy. Am. J. Enol. Vitic..

[75] Lawless H.T., Heymann H. (2010). Sensory Evaluation of Food: Principles and Practices.

[76] Wakeling I.N., MacFie H.J.H. (1995). Designing consumer trials balanced for first and higher orders of carry-over effect when only a subset of k samples from t may be tested. Food Qual. Prefer..

[77] Lawless H.T., Liu Y.F., Goldwyn C. (1997). Evaluation of Wine Quality Using a Small-Panel Hedonic Scaling Method. J. Sens. Stud..

[78] Gay C. (1998). Invitation to comment. Food Qual. Prefer..

[79] Yeomans K., Golder P. (1982). The Guttman-Kaiser criterion as a predictor of the number of common factors. J. R. Stat. Soc. Ser. D.

[80] Costello A.B., Osborne J.W. (2005). Best Practices in Exploratory Factor Analysis: Four Recommendations for Getting the Most From Your Analysis. Pract. Assess. Res. Eval..

[81] Lê S., Husson F. (2008). Sensominer: A package for sensory data analysis. J. Sens. Stud..

[82] Husson F., Josse J., Lê S., Mazet J. FactoMineR: Multivariate Exploratory Data Analysis and Data Mining with R 2012. http://cran.r-project.org/package=FactoMineR.

[83] Fellows I. (2012). Deducer: A Data Analysis GUI for R. J. Stat. Softw..

[84] De Mendiburu F. Agricolae: Statistical Procedures for Agricultural Research 2013. http://cran.r-project.org/package=agricolae.

